# Surface Engineering of NK Cells with Poly-L-Glutamic Acid Enhances Tumor-Selective Immunotherapy Against Ovarian Cancer

**DOI:** 10.3390/cells15090800

**Published:** 2026-04-28

**Authors:** Yoonbum Park, Ashok Kumar Jangid, Kyung Mu Noh, Eunha Kim, Chae Eun Lee, Kyobum Kim

**Affiliations:** 1 Department of Chemical & Biochemical Engineering, Dongguk University, Seoul 04620, Republic of Korea; dean4528@dgu.ac.kr (Y.P.); ashok4483@gmail.com (A.K.J.); kyungmu.noh@dgu.ac.kr (K.M.N.); kim4710@dgu.ac.kr (E.K.); jum0051@dgu.ac.kr (C.E.L.); 2 Cellbastian Inc., 30, Pildong-ro 1-gil, Jung-gu, Seoul 04620, Republic of Korea

**Keywords:** natural killer cells, membrane engineering, ovarian cancer, poly-L-glutamic acid, tumor targeting, immunotherapy

## Abstract

**Highlights:**

**What are the main findings?**

- Lipid-mediated surface engineering of NK cells with poly-L-glutamic acid (PLE) significantly enhanced effector–target interactions with ovarian cancer cells via cholesterol-associated membrane binding.
- PLE-coated NK cells exhibited markedly improved tumor-specific cytotoxicity without increasing off-target killing against normal fibroblasts.

**What are the implications of the main findings?**

- This receptor-independent membrane targeting strategy provides a versatile approach to enhance NK cell recognition of solid tumors.
- PLE-based surface engineering offers a simple and scalable platform to improve the efficacy and selectivity of NK cell-based cancer immunotherapy.

**Abstract:**

Natural killer (NK) cells are promising effectors for cancer immunotherapy, as they can recognize and eliminate tumor cells without prior antigen sensitization. However, insufficient tumor recognition remains a critical limitation that reduces the anticancer efficacy of NK cells against solid tumors. To address this limitation, we developed a lipid-mediated cell membrane engineering strategy to enhance the targeting and cytotoxic efficacy of NK cells toward solid tumors, particularly ovarian cancer cells. In this strategy, poly-L-glutamic acid (PLE) was employed as an ovarian cancer-targeting module due to the specific affinity of PLE for cholesterol-rich membrane domains. To display PLE on NK cells, a lipid moiety is incorporated to anchor PLE onto the NK cell membrane via hydrophobic insertion, enabling rapid and non-genetic surface modification. As a result, the surface-engineered NK cells with PLE-Lipid (i.e., PLE-NK) displayed PLE on the NK cell surface, allowing direct recognition of ovarian cancer cells without compromising the intrinsic properties of NK cells. This enhanced recognition subsequently increased NK–cancer cluster formation by promoting interactions between membrane-presented PLE on NK cells and cholesterol on ovarian cancer cells. Consequently, PLE-NK cells exhibited enhanced cytotoxicity against ovarian cancer cells (i.e., OVCAR-3 cells) and effectively disrupted 3D tumoroids, while PLE-NK cells showed no off-target effects on normal fibroblasts. Collectively, these findings demonstrate that PLE-Lipid-mediated NK surface engineering provides a simple and effective strategy to improve the tumor targeting ability of NK cells and offers a promising platform for NK cell-based immunotherapy against ovarian cancer.

## 1. Introduction

Ovarian cancer is the fifth leading cause of cancer-related mortality among women worldwide while remaining one of the most difficult gynecologic malignancies to treat. Despite advances in cytoreductive surgery and systemic chemotherapy, overall survival remains poor [1]. The high mortality of ovarian cancer is largely attributed to three major clinical challenges. First, early detection remains difficult, as early-stage disease often presents with minimal or nonspecific symptoms that are frequently misattributed to benign gastrointestinal or menopausal conditions [2]. Second, most cases are epithelial ovarian cancers, most commonly high-grade serous ovarian carcinoma (HGSOC), an aggressive subtype characterized by rapid peritoneal dissemination that renders the complete eradication of microscopic residual disease clinically challenging [3]. Finally, although the standard treatment consists of cytoreductive surgery followed by platinum–taxane-based chemotherapy [4], high relapse rates and the development of platinum resistance remain major obstacles to long-term survival [5].

In recent years, antibody–drug conjugates (ADCs) have demonstrated clinical benefits in platinum-resistant ovarian cancer (PROC) [6]. However, the efficacy of ADCs is inherently limited by the reliance on predictive biomarkers and the heterogeneous expression of target antigens, such as FOLR1 and MSLN [7,8]. Furthermore, resistance mechanisms, including reduced antigen expression, impaired internalization, and defective intracellular trafficking, could restrict payload delivery and diminish therapeutic efficacy [9].

These limitations have prompted the development of alternative immunotherapeutic strategies, particularly adoptive cell therapy (ACT). Among various immune cell players, natural killer (NK) cells have been intensively investigated due to their intrinsic antitumor therapeutic efficacy. NK cells do not require antigen-specific priming and can directly recognize and eliminate tumor cells without prior antigen-presenting cell-dependent sensitization [10]. Especially, NK cells discriminate between normal and malignant cells through “missing-self” and “induced-self” recognition. Missing-self recognition occurs when tumor cells downregulate MHC class I molecules, thereby reducing inhibitory signaling and permitting NK cell activation [11]. In addition, activating receptors, such as NKG2D, which bind stress-inducible ligands that include MICA, MICB, and ULBPs expressed on transformed cells, mediate induced-self recognition [12]. Once activated, NK cells form an immunological synapse and induce apoptosis primarily via perforin–granzyme-mediated cytotoxicity, a process dependent on stable cell–cell interactions [13]. Based on these characteristics, NK cells can be applied as allogeneic cell therapeutics and have entered clinical investigation for cancer treatment [14]. However, within the solid tumor microenvironment, downregulation of activating ligands and upregulation of inhibitory signals can impair NK cell function and promote immune evasion [15]. Moreover, insufficient expression of tumor-targeting receptors on NK cells may limit their ability to efficiently recognize certain cancer cells [16]. To overcome this limitation, ex vivo cell surface engineering has emerged as a strategy to enhance the tumor-targeting capacity of immune cells [17,18,19,20,21,22,23]. This approach involves the attachment of synthetic ligands onto the NK cell membrane to confer the augmented capability of tumor antigen recognition, without genetic modification [24,25,26].

In this study, we developed a lipid-based surface engineering strategy to enhance NK cell targeting toward ovarian cancer cells. Poly-L-glutamic acid (PLE) was selected as a functional ligand based on its reported affinity toward tumor cells [27,28,29]. Specifically, recent studies suggest that PLE can associate with cancer cell membranes through interactions with cholesterol-rich lipid raft domains [30]. In addition, PLE is a biocompatible, non-immunogenic, and biodegradable polymer [31]. Notably, epithelial ovarian cancer cells, especially HGSOC, exhibit dysregulated cholesterol metabolism and an increased abundance of lipid raft structures [32,33], which may facilitate such interactions with PLE-modified nanoparticles [29]. Another suggested binding mechanism of PLE on tumor cell surfaces is that its carboxylated structure possesses mucoadhesive properties that facilitate interactions with mucins [34,35], which are frequently overexpressed in ovarian cancer [36]. Although these binding mechanisms of PLE on tumor cell surfaces have been suggested, it remains unclear which factor predominantly contributes to PLE-mediated cancer targeting. However, when cholesterol was extracted from the cancer cell membranes, the binding affinity of PLE-modified nanoparticles was reduced [30]. Collectively, these properties make PLE a suitable ligand for NK cell surface modification to improve tumor targeting.

**Scheme 1:**
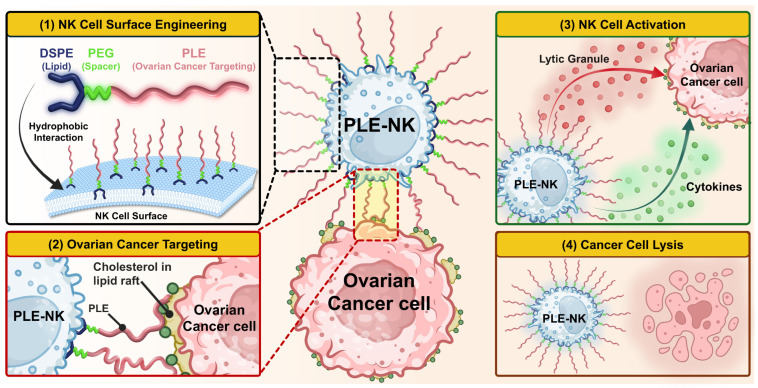
Schematic illustration of PLE-Lipid conjugate-mediated NK cell surface engineering strategy and subsequent tumor recognition, activation, and cytotoxic responses of PLE-NK cells against ovarian cancer.

To implement this strategy, we synthesized a PLE-poly ethylene glycol (PEG) 2k-1,2-distearoyl-sn-glycero-3-phosphoethanolamine (DSPE) (i.e., PLE-Lipid) conjugate composed of three functional components: (1) DSPE as a hydrophobic lipid anchor for NK cell membrane insertion, (2) PEG as a hydrophilic blocking layer to reduce nonspecific adsorption and uptake, and (3) PLE as an ovarian cancer-targeting moiety. As illustrated in Scheme 1, this PLE-Lipid conjugate enables rapid NK cell surface engineering and subsequent tumor targeting. Within 30 min incubation of NK cells with the PLE-Lipid conjugate, the DSPE lipid tail spontaneously inserts into the plasma membrane via hydrophobic self-assembly, thereby displaying PLE on the NK cell surface (i.e., PLE-NK) to enhance ovarian cancer recognition. This NK membrane engineering is facile and rapid and yields uniformly modified NK cells, without requiring genetic engineering or extensive ex vivo expansion. Importantly, this NK surface modification did not deteriorate NK cell viability or major receptor expression. Moreover, surface-engineered NK cells exhibited enhanced binding to ovarian cancer cells (i.e., OVCAR-3), mediated by interactions between PLE on NK cells and cholesterol-rich compartments of cancer cells, which consequently augmented tumor cell lysis. Therefore, these findings suggest that lipid-based NK cell surface engineering represents a promising strategy to enhance tumor targeting and cytotoxic efficacy in NK cell-mediated immunotherapeutic applications.

## 2. Materials and Methods

### 2.1. Synthesis of the Lipid-PLE Biomaterials

PLE sodium salt (Sigma-Aldrich, St. Louis, MO, USA) and DSPE-PEG-COOH (Sigma-Aldrich, St. Louis, MO, USA) were used as the lipid conjugate component. PLE-Lipid conjugates were synthesized using 1-ethyl-3-(3-dimethylaminopropyl)carbodiimide (EDC, Sigma-Aldrich, St. Louis, MO, USA)/N-hydroxysuccinimide (NHS, Sigma-Aldrich, St. Louis, MO, USA)-mediated coupling. DSPE-PEG-COOH was dissolved in anhydrous dimethylformamide (DMF, Sigma-Aldrich, St. Louis, MO, USA) and activated with EDC and NHS at room temperature (RT). PLE was dissolved in phosphate-buffered saline (PBS, Sigma-Aldrich, St. Louis, MO, USA), and the activated DSPE-PEG-COOH solution was added to the PLE solution. 4-Dimethylaminopyridine (DMAP, Sigma-Aldrich, St. Louis, MO, USA) was added as a catalyst, and the reaction mixture was stirred at RT for 24 h. The reaction product was purified by dialysis against distilled water for 24 h and subsequently lyophilized. Successful conjugation of DSPE-PEG to PLE was confirmed by ^1^H-NMR (500 MHz FT-NMR spectrometry, Bruker, Bremen, Germany) and FT-IR spectroscopy (PerkinElmer FT-IR Spectrum Two, PerkinElmer, Springfield, IL, USA). For fluorescence-based characterization of PLE-coated NK cells, DSPE-PEG-PLE (PLE-Lipid) was conjugated to Alexa Fluor^TM^ 488 hydrazide (Invitrogen, Carlsbad, CA, USA) via EDC/NHS coupling, as previously described. The resulting conjugate is referred to as PLE-Lipid-FL.

### 2.2. Cell Culture

NK-92MI cells (American Type Culture Collection, ATCC, Manassas, VA, USA) were used as effector cells and cultured in Minimum Essential Medium Alpha (MEMα; Gibco, Grand Island, NY, USA) supplemented with 12.5% fetal bovine serum (FBS, Gibco, Grand Island, NY, USA), 12.5% horse serum (Gibco, Grand Island, NY, USA), 1% penicillin–streptomycin solution (Corning, Corning, NY, USA), 0.2 mM myo-inositol (Sigma-Aldrich, St. Louis, MO, USA), 0.1 mM β-mercaptoethanol (Sigma-Aldrich, St. Louis, MO, USA), and 0.02 mM folic acid (Sigma-Aldrich, St. Louis, MO, USA). NIH: OVCAR-3 cells (target cells) were obtained from the Korean Cell Line Bank (KCLB, Seoul, Republic of Korea). OVCAR-3 cells were maintained in 89% of RPMI 1640 Medium (Gibco, Grand Island, NY, USA), supplemented with 20% heat-inactivated FBS (Gibco, Grand Island, NY, USA) and 1% penicillin–streptomycin solution (Corning, Corning, NY, USA). Human fibroblasts (Lonza, Basel, Switzerland) were cultured in growth medium consisting of 89% Dulbecco’s modified Eagle’s medium (DMEM, Corning, Corning, NY, USA), 10% FBS (Gibco, Grand Island, NY, USA), and 1% penicillin–streptomycin solution (Corning, Corning, NY, USA). All cells were cultured at 37 °C in a humidified incubator with 5% CO_2_.

### 2.3. Surface Engineering and Characterization of NK Cells

NK-92MI cells (5 × 10^5^ cells) were centrifuged and incubated with 100 μL of PLE-Lipid dissolved in basal MEMα medium (Gibco, Grand Island, NY, USA) at RT for 30 min. After incubation, cells were washed twice with basal medium to remove unbound materials. For coating concentration optimization, NK cells were coated with PLE-Lipid-FL (0–3 mg/mL), as above. Then, the surface-engineered NK cells were characterized using flow cytometry (Beckman Coulter, Brea, CA, USA). Additionally, PLE-NK cells prepared at the optimal coating concentration were observed by fluorescence microscopy (Ti-E, Nikon, Tokyo, Japan), and the resulting images were analyzed using ImageJ software 1.53e.

### 2.4. Cell Coating Maintenance

The surface-engineered NK cells were prepared using 2 mg/mL of PLE-Lipid-FL solution, and 5 × 10^5^ cells were prepared for each time point (0, 0.5, 1, 1.5, 2, 3, 7.5, and 17.5 h). All samples were incubated under standard culture conditions (37 °C, 5% CO_2_). At each designated time point, remaining PLE-Lipid-FL on NK cells was analyzed by flow cytometry. Coating stability was assessed by comparing mean fluorescence intensity (MFI).

### 2.5. Coating Efficacy

To quantify the amount of coating material associated with cells, the fluorescence intensity of NK cell lysates was measured after membrane coating. Briefly, surface-engineered NK cells (1 × 10^6^ cells) were prepared using PLE-Lipid-FL solution (2 mg/mL in basal medium). Then, NK cells were lysed with 300 μL RIPA buffer (Elpis-Biotech, Daejeon, Republic of Korea) at 4 °C for 30 min. The lysates were diluted 1:1 with basal medium, and 100 μL of solution was transferred to a 96-well plate. Fluorescence intensity was measured using a SpectraMax iD3 microplate reader (Ex/Em = 485/535 nm/nm, Molecular Device, Sunnyvale, CA, USA). The measured fluorescence intensity was quantified by comparison with a standard curve.

### 2.6. Viability and Proliferation Capacity of NK Cells

To rule out potential cytotoxic effects of PLE-Lipid, the viability of PLE-NK cells coated with various PLE-Lipid concentrations (0, 0.5, 1.0, 2.0, and 3.0 mg/mL) was measured over time after coating NK cells for 30 min. The coated NK cells (5 × 10^4^ cells per well) were seeded into 96-well culture plates and were incubated for 48 h. Cell viability was assessed at 0, 24, and 48 h using a WST-1 assay (EZ-Cytox, DoGenBio, Seoul, Republic of Korea). Following the addition of EZ-Cytox reagent, the well plates were incubated at 37 °C for 3 h, and absorbance was measured at 450 nm using a microplate reader. Cell viability at 24 and 48 h was normalized to unmodified NK cells at 0 h. Based on these results, 2 mg/mL was determined to be the highest polymer concentration that did not affect NK cell viability with proliferation, and NK cells coated under this condition for 30 min were subsequently employed for all further experiments.

### 2.7. Surface Ligand/Receptor Availability

The availability of PLE-NK cell surface ligands (i.e., FasL and TRAIL), which are closely related to apoptosis induction in target cells, was assessed. NK cells and PLE-NK cells were incubated with APC-conjugated FasL and TRAIL antibodies (BD Biosciences, Franklin Lakes, NJ, USA) at 4 °C for 30 min. After incubation, the cells were washed twice with cold PBS and analyzed using flow cytometry.

### 2.8. E:T Cluster Formation

Effector-to-target (E:T) cluster formation was evaluated to assess the physical interaction between NK cells and ovarian cancer cells. The human ovarian cancer cell line OVCAR-3 was stained with 1 μM CellTracker^TM^ Red CMTPX Dye (Invitrogen, Carlsbad, CA, USA) and subsequently washed twice with basal medium. Simultaneously, NK cells were stained with 0.5 μM Calcein-AM (Invitrogen, Carlsbad, CA, USA) at 37 °C for 30 min and washed twice with basal medium. PLE-NK cells were prepared using the calcein-stained NK cells as previously described. Subsequently, stained NK or PLE-NK cells were co-incubated with target cells at an E:T ratio of 1:1 (total 6 × 10^5^ cells) in a 1.5 mL microcentrifuge tube at 37 °C for 30 min. E:T clusters were identified by flow cytometric analysis based on dual fluorescence signals originating from both effector (green) and target (red) cells. Cells emitting both green and red fluorescence were selectively gated and quantified as a percentage of the total detected cellular events. For the cholesterol-extracted model, OVCAR-3 cells were treated with methyl-β-cyclodextrin (MβCD, Sigma-Aldrich, St. Louis, MO, USA) dissolved in complete medium at a concentration of 12.8 mg/mL for 4 h to generate cholesterol-extracted ovarian cancer cells. To assess the specificity of E:T cluster formation, PLE-NK cells were co-incubated with normal fibroblasts under identical conditions.

### 2.9. Anticancer Assay

Anticancer efficacy of PLE-NK cells was evaluated using a calcein release assay. In this study, OVCAR-3 human ovarian cancer cells were used as target cells to assess the effect of PLE-NK cell-mediated cytotoxicity. In addition, human fibroblasts were used as a non-cancerous control target to assess the absence of nonspecific cytotoxicity. 1 × 10^4^ target cells (OVCAR-3 cells or fibroblasts) were stained with 15 μM Calcein-AM (Invitrogen, Carlsbad, CA, USA) at 37 °C for 30 min and washed twice with basal medium. Then, calcein-labeled target cells were co-cultured with unmodified NK cells or PLE-NK cells at a 10:1 E:T ratio in a 96-well plate and incubated at 37 °C for 4 h. Following co-culture, fluorescence intensity of the supernatant was measured by a microplate reader (Ex/Em = 485/535 nm/nm).

The percentage of specific cell lysis was calculated using the following equation:

Specific lysis (%) = (test release − spontaneous release)/(maximum release − spontaneous release) × 100, where spontaneous release was defined as the fluorescence intensity of calcein-labeled target cells cultured without effector cells and maximum release was obtained by treating target cells with 5% Triton X-100 (Sigma-Aldrich, St. Louis, MO, USA).

### 2.10. Quantification of Cytokine

To quantify the secreted cytokines (TNF-*α* and IFN-*γ*) and lytic granule (granzyme B), NK or PLE-NK cells (1 × 10^5^ cells) were co-cultured with OVCAR-3 cells at a 10:1 E:T ratio and incubated at 37 °C for 4 h. The centrifuged supernatants were collected, and cytokines (TNF-*α* and IFN-*γ*) and cytotoxic granule (granzyme B) levels were measured by ELISA (PeproTech, Cranbury, NJ, USA or Abcam, Cambridge, UK) following the manufacturers’ protocols.

### 2.11. Spheroid Formation and Anticancer Testing

OVCAR-3 cells were stained with 10 μM CellTracker^TM^ Red CMTPX Dye (Invitrogen, Carlsbad, CA, USA). Three-dimensional (3D) spheroids were generated by seeding 5 × 10^3^ OVCAR-3 cells onto agarose-coated 96-well plates (50 μL of 1.5% (*w*/*v*) agarose per well) and were incubated at 37 °C for 24 h. NK or PLE-NK cells (5 × 10^4^ cells) were then applied to the OVCAR-3 spheroids and incubated for an additional 24 h. Following incubation, spheroid disruption was observed by fluorescence microscopy, and acquired images were analyzed using ImageJ software.

### 2.12. Statistical Analysis

All quantitative data are expressed as the mean ± standard deviation (SD, *n* = 3) and analyzed by a *t*-test or one-way analysis of variance (ANOVA) with Tukey’s post hoc method. Differences were considered statistically significant at *p* values of <0.05. All statistical analyses were performed using GraphPad Prism 8.0.2 (GraphPad Software Inc, La Jolla, CA, USA).

## 3. Results

### 3.1. Characterization of PLE-Lipid and PLE-NK Cells

**Figure 1:**
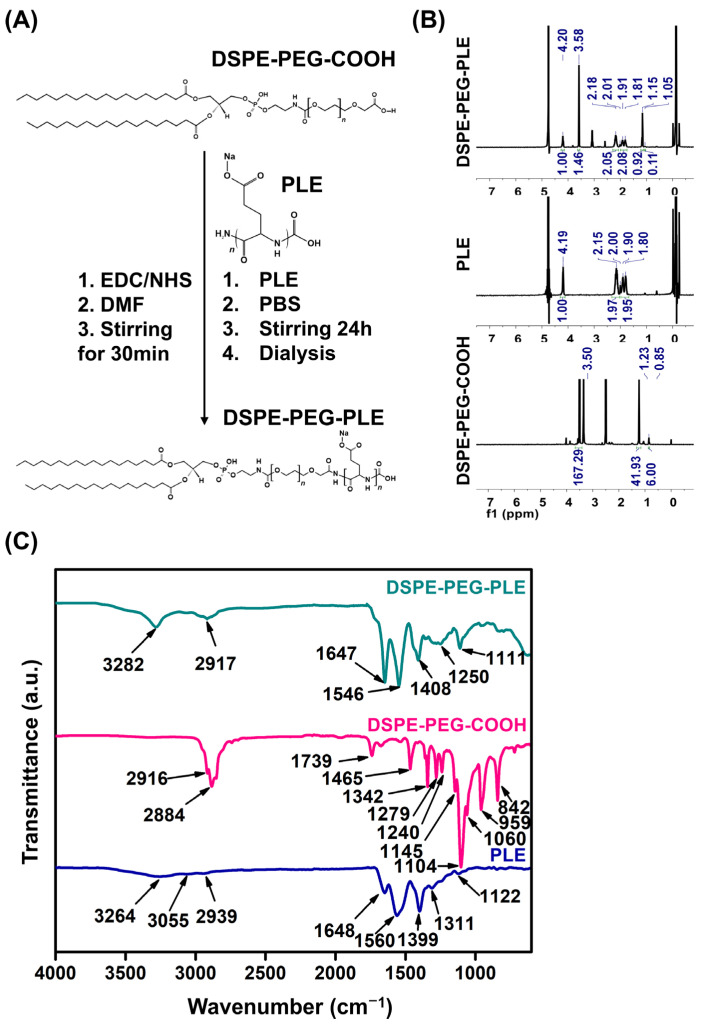
Synthesis and characterization of PLE-Lipid. (**A**) Method for synthesizing PLE-Lipid via EDC/NHS coupling. (**B**) The formation of PLE-Lipid was verified through ^1^H-NMR (**C**) FT-IR spectra of PLE, DSPE-PEG, and the synthesized DSPE-PEG-PLE conjugate, confirming the amide bond formation between DSPE-PEG and PLE.

To engineer NK cell surfaces with PLE-Lipid, DSPE-PEG-COOH was conjugated to PLE via EDC/NHS-mediated amide coupling reaction (Figure 1A). The resulting PLE-Lipid consists of two functional components, including (1) a DSPE lipid moiety enabling stable NK surface anchoring via hydrophobic interaction with plasma membrane layers [37] and (2) a PLE segment for ovarian cancer cell targeting [30]. The carboxyl group of DSPE-PEG-COOH was activated using EDC and NHS to generate an NHS ester intermediate, which subsequently reacted with amine groups of PLE to form an amide bond. Figure 1A shows the synthetic process of the PLE-Lipid biomaterial, while Figure 1B,C depict the molecular structure confirmation using NMR and FT-IR. The successful conjugation of the DSPE-PEG-COOH with PLE was first confirmed by NMR analysis, as demonstrated in Figure 1B. The synthesized PLE-Lipid shows the characteristic peaks at δ 3.5 ppm that belongs to the CH_2_CH_2_O protons of PEG chains; that at δ 1.05–1.18 ppm belongs to DSPE lipid hydrocarbon chains, while that at δ 2.05–2.18 ppm belongs to PLE signals. The presence of all the characteristic peaks of DSPE-PEG and PLE in the PLE-Lipid conjugate demonstrates the successful formation of PLE-Lipid via amide bond formation. Furthermore, the amide bond formed between DSPE-PEG and PLE was confirmed by FT-IR analysis. After conjugation, the FT-IR spectrum shows 3282 cm^−1^ representing amide bond N-H stretching, 2917 cm^−1^ C-H stretching of lipid units, 1546 and 1647 cm^−1^ confirming amide bond formation, 1408–1250 cm^−1^ representing C-N and PEG stretching vibrations, and 1111 cm^−1^ bending vibrations (Figure 1C). The appearance of amide peaks and the combined features of PEG, DSPE, and PLE confirm the successful covalent conjugation of PLE to DSPE-PEG via the EDC/NHS coupling mechanism.

### 3.2. Biomaterial-Mediated Ex Vivo NK Cell Surface Engineering

**Figure 2:**
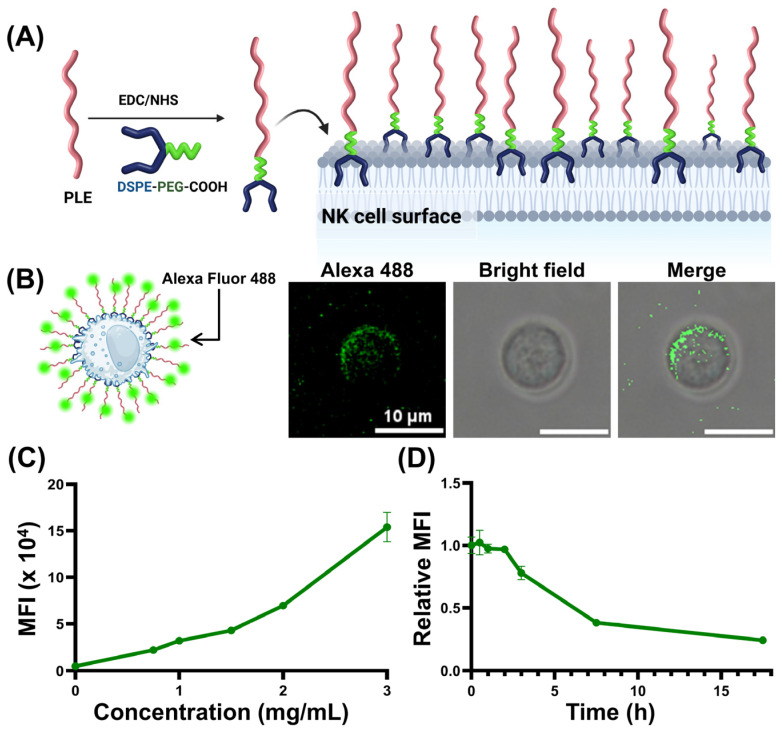
Characterization of surface-engineered NK cells. (**A**) Schematic illustration of PLE-Lipid synthesis via EDC/NHS-mediated coupling reaction and its application in NK cell surface engineering. (**B**) Optical and fluorescence microscopy images of NK cells coated with PLE-Lipid-FL. (**C**) Flow cytometry analysis of coating efficiency at varying PLE-Lipid concentrations. (**D**) Flow cytometry analysis of coating retention on NK cells over time. All quantitative results are presented as mean ± SD (*n* = 3).

To enable the biomaterial-mediated engineering of NK cell membranes, an amphiphilic PLE-Lipid was synthesized and used for surface functionalization, as illustrated in Figure 2A. To enable visualization and quantitative analysis of the NK cell surface engineering through the membrane immobilization of PLE-Lipid, PLE-Lipid was fluorescently labeled with Alexa Fluor^TM^ 488 (PLE-Lipid-FL). NK cells were then incubated with PLE-Lipid-FL solution for 30 min and subsequently analyzed through fluorescence microscopy and flow cytometry. Fluorescence microscopy images indicated clear membrane-associated fluorescence signals on the coated NK cells (Figure 2B). To further evaluate concentration-dependent coating efficiency, engineered NK cells were analyzed by flow cytometry at increasing concentrations of PLE-Lipid-FL. As a result, a corresponding increase in MFI was observed (Figure 2C).

To quantify the amount of membrane-anchored PLE-Lipid, the fluorescence intensity of lysates from PLE-Lipid-FL-coated NK cells was measured and quantified using a standard calibration curve generated from a known concentration of PLE-Lipid-FL [38]. The coating amount was determined to be 25.56 ng per 10^5^ NK cells. To further assess the stability of membrane-anchored PLE-Lipid over time, 5 × 10^5^ NK cells coated with PLE-Lipid-FL (2 mg/mL) were cultured, and membrane-associated fluorescence intensity was analyzed by flow cytometry at the indicated time points [37]. MFI remained close to the 0 h value during the first 2 h (i.e., approximately 100%), gradually decreased to approximately 40% by 8 h, and remained detectable at 20 to 25% up to 17 h post-coating. These results demonstrate stable membrane association of PLE-Lipid over a biologically relevant temporal time window (Figure 2D).

### 3.3. Intrinsic Properties of NK Cells Coated with PLE-Lipid

**Figure 3:**
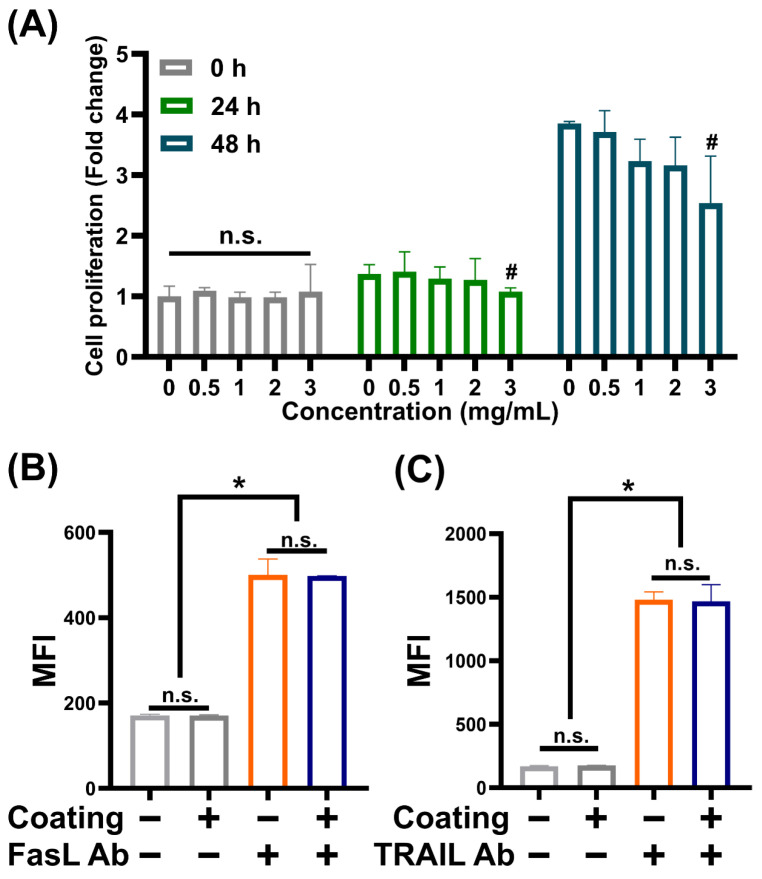
Effect of coating on NK cell viability and function. (**A**) WST-1 assay to assess the proliferation capacity of PLE-Lipid-coated NK cells. (**B**) FasL and (**C**) TRAIL ligand on PLE-Lipid-engineered NK cells measured via flow cytometry. # indicates significant differences (*p* < 0.05) as compared to 0 mg/mL groups within the same time point; all quantitative results are presented as mean ± SD (*n* = 3). * *p* < 0.05. “n.s.” indicates statistically non-significant.

The working concentration of PLE-Lipid without cytotoxic influence toward NK cells was then validated (Figure 3A). Here, NK cells were incubated with varying concentrations of PLE-Lipid, and cell viability was analyzed using a WST-1 assay. As a result, NK cell viability remained stable up to 2 mg/mL at 0, 24, and 48 h. In contrast, treatment with 3 mg/mL of PLE-Lipid resulted in a statistically significant reduction in viability compared to the control group at both 24 and 48 h. Therefore, 2 mg/mL of PLE-Lipid solution was selected as the optimal coating concentration, providing efficient NK membrane coating without significant cytotoxicity, and it was used for subsequent functional experiments.

Next, to assess the effect of surface engineering on the intrinsic signaling of NK cells, the surface expression of membrane-bound death receptor ligands (FasL and TRAIL) was analyzed by flow cytometry [24]. The results in Figure 3B,C indicate that PLE-Lipid coating did not significantly alter the surface ligand accessibility of either FasL or TRAIL compared to unmodified NK control [39].

### 3.4. Target Recognition and Anticancer Efficacy of PLE-NK Cells

#### 3.4.1. Enhanced Target Recognition of PLE-NK Cells

**Figure 4:**
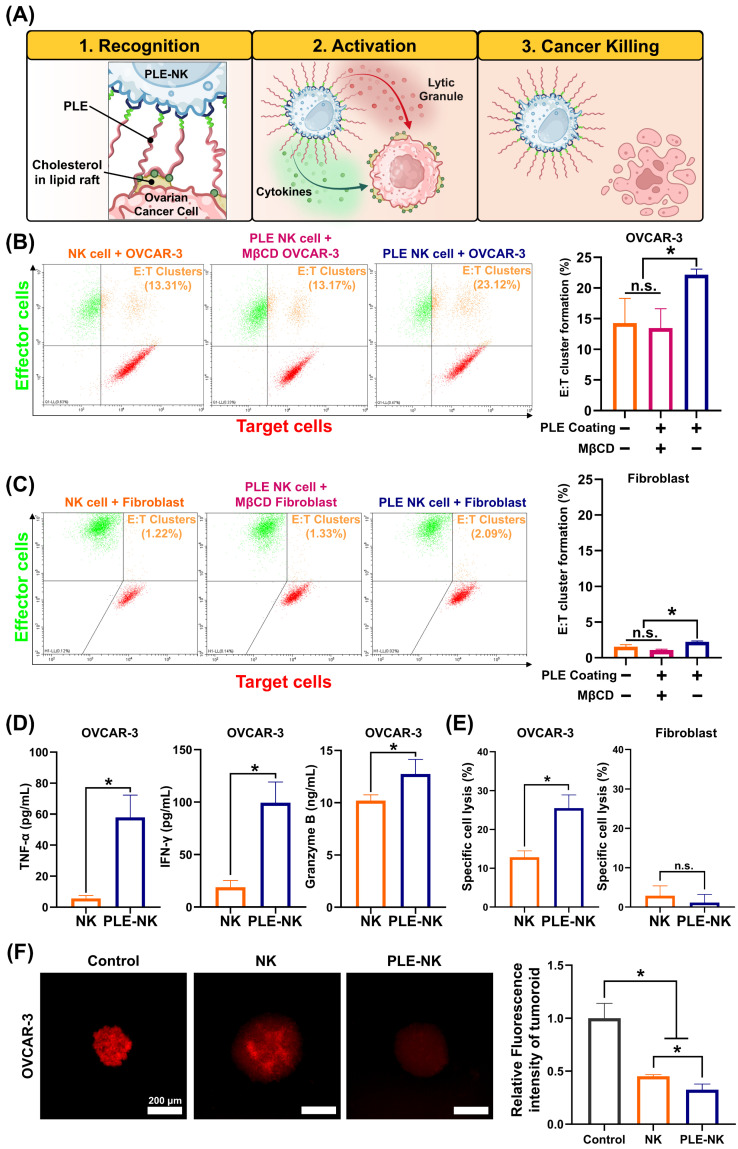
Evaluation of target recognition and anticancer ability of PLE-NK. Quantitative and representative flow cytometric analysis of cholesterol-dependent target recognition ability of PLE-NK cells. (**A**) Schematic illustration of the enhanced tumor recognition and cytotoxicity mediated by PLE-Lipid-engineered NK cells. (**B**) OVCAR-3 cells were co-cultured with NK or PLE-NK cells for 30 min and analyzed by flow cytometry. Double-positive events (Calcein^+^/Cell Tracker Red^+^) represent effector-to-target (E:T) cluster formation. (**C**) E:T cluster formation with normal fibroblasts as control cells. (**D**) Cytokines (TNF-α, IFN-γ) and cytotoxic granule (granzyme B) secretion by NK cells and PLE-NK cells against OVCAR-3 cells. Protein levels were measured by ELISA using supernatants collected from NK–target co-culture media for 4 h. (**E**) Percentage of specific cell lysis of target cells by NK cells or PLE-NK cells. Effector cells were co-cultured with OVCAR-3 cells or normal fibroblasts at an E:T ratio of 10:1 for 4 h. Cytotoxicity was quantified using a calcein release assay. (**F**) Anticancer functionality of NK cells against OVCAR-3 3D tumoroid. Prior to tumoroid formation, OVCAR-3 cells were stained with CellTracker^TM^ Red CMTPX Dye. NK or PLE-NK cells (5 × 10^4^) were added to the spheroids and incubated for 24 h. Fluorescent intensity was evaluated using ImageJ software. All quantitative results are presented as mean ± SD (*n* = 3). * *p* < 0.05. “n.s.” indicates statistically non-significant.

Figure 4A illustrates the mechanism by which PLE-NK cells exhibit improved tumor recognition through interactions with cholesterol-rich membrane domains, leading to enhanced cytokines and cytotoxic granule release [30], which ultimately results in enhanced ovarian cancer cell killing. Effective NK cell-mediated cytotoxicity requires stable physical engagement between effector and target cells, which precedes immunological synapse formation and subsequent granule release. Therefore, we examined whether PLE-mediated surface modification enhances NK–ovarian tumor cell interactions [40]. Elevated interaction between PLE-coated NK cells and OVCAR-3 target cancer cells were analyzed by E:T cluster formation using flow cytometry [26]. For this analysis, NK cells were labeled with calcein (green), while OVCAR-3 target cells were counter-stained with CellTracker^TM^ Red, prior to co-incubation. E:T cluster formation was then quantified by flow cytometry, based on double-positive (green/red) events indicating the formation of NK–cancer complexes. PLE-NK cells exhibited significantly increased E:T cluster formation (22.17%) compared to unmodified control NK cells (14.24%), representing an approximately 1.6-fold increase in tumor cell engagement (Figure 4B) [38].

This E:T cluster formation primarily relies on the specific molecular interaction between NK cell membrane-immobilized PLE and cholesterol compartments located on target tumor cells. To investigate this specific recognition procedure, target OVCAR-3 cells were pre-treated with MβCD to deplete surface cholesterols prior to co-incubation with PLE-NK cells. MβCD selectively extracts cholesterol from the plasma membrane by forming inclusion complexes with cholesterol molecules, thereby disrupting cholesterol-rich lipid raft domains [41,42]. Figure 4B shows that cholesterol depletion markedly reduced the E:T cluster formation of PLE-NK cells (13.45%), restoring the interaction level to that of control NK cells. These results demonstrate that the PLE-mediated tumor recognition of surface-engineered NK cells is regulated by interactions with cholesterol-rich membrane domains in target cancer cells.

To further evaluate whether PLE-mediated surface modification induces nonspecific interactions with non-cancerous cells, we performed an additional E:T cluster formation assay using fibroblasts as target cells while maintaining identical experimental conditions. In this experiment, fibroblasts were stained with CellTracker^TM^ Red and co-incubated with calcein-labeled NK or PLE-NK cells following the same protocol used for ovarian cancer cells. E:T cluster formation was subsequently quantified by flow cytometry based on double-positive events representing NK–target cell complexes. Figure 4C shows that fibroblasts exhibited minimal E:T cluster formation (approximately 2%) across all experimental groups, which was significantly lower than that observed with ovarian cancer cells. These results indicate that, under the same conditions, NK cell binding to fibroblasts occurs at negligible levels.

Interestingly, PLE-NK cells showed a slight increase in cluster formation with fibroblasts compared to unmodified NK cells. This effect may be attributed to the presence of cholesterol-rich lipid raft domains in fibroblast membranes [43], which could provide limited binding interfaces for PLE-mediated interactions. However, overall binding remained substantially lower than that observed with ovarian cancer cells. Furthermore, as shown in Figure 4E, these limited interactions did not result in increased cytotoxic activity against fibroblasts, indicating that recognition of normal cells does not trigger NK cell-mediated cytotoxic activation.

#### 3.4.2. Enhanced Anticancer Activity of PLE-NK Cells

As augmented effector–target interactions subsequently facilitate immunological synapse formation and the localized delivery of cytotoxic granules, we further evaluated whether increased cluster formation could be translated into enhanced NK cell activation and improved tumor cell killing of PLE-NK cells [26]. To assess NK cell activation following target recognition, NK cells were co-cultured with OVCAR-3 cells at a 10:1 E:T ratio for 4 h, and TNF-α secretion was measured by ELISA. PLE-NK cells exhibited a significantly higher level of TNF-α secretion (57.94 pg/mL) compared to unmodified NK cells (5.75 pg/mL), indicating enhanced activation and cytotoxic potential driven by improved tumor recognition. Consistently, PLE-NK cells also showed increased IFN-γ secretion (99.4 pg/mL), whereas unmodified NK cells produced 18.9 pg/mL, further supporting enhanced immunoregulatory activity. In addition, granzyme B secretion was modestly elevated in PLE-NK cells (12.75 pg/mL), while unmodified NK cells exhibited a level of 10.20 pg/mL, indicating enhanced granule-mediated cytotoxic potential (Figure 4D).

When NK cells were co-cultured with OVCAR-3 cells for 4 h with a 10:1 E:T ratio, PLE-NK cells exhibited significantly higher specific cell lysis than unmodified NK cells (Figure 4E). In particular, PLE-NK cells induced 25.5% specific lysis of OVCAR-3 cells compared to 12.8% lysis by unmodified NK cells, representing a nearly 2-fold increase in cytotoxic activity by membrane presentation of PLE onto NK cells. Importantly, no significant increase in cytotoxicity was observed against normal fibroblasts, confirming tumor-selective enhancement, rather than nonspecific toxicity.

This enhanced antitumor efficacy of PLE-NK cells toward ovarian cancer cells was further confirmed using a physiologically 3D tumoroid model [25]. CellTracker^TM^ Red-prelabeled OVCAR-3 cell 3D tumoroids were incubated with NK or PLE-NK cells for 24 h to evaluate their anticancer efficacy. After 24 h of co-incubation, fluorescence imaging revealed substantial disruption of tumoroids in the PLE-NK group compared to the control and unmodified NK cell groups (Figure 4F). Quantitative analysis of fluorescence intensity also demonstrated a significant reduction in spheroid signal in the PLE-NK group, consistent with enhanced tumor cell killing. Therefore, our results collectively demonstrate that PLE-mediated surface modification could improve the tumor targeting capability of NK cells and enhance cytotoxic efficacy against ovarian cancer cells, particularly within a 3D tumoroid model.

## 4. Discussion

In this study, we developed a cellular membrane engineering strategy to enhance the recognition and cytotoxic efficacy of NK cells against ovarian cancer. By introducing PLE-Lipid conjugates onto the NK cell surface, we aimed to augment NK–tumor interactions at the immune synapse interfaces, without altering the intrinsic properties of NK cells. The amphiphilic lipid–polymer design enables rapid membrane insertion through hydrophobic and lipophilic interactions between the DSPE lipid tails of biomaterials and the phospholipid bilayer of cell surfaces, resulting in stable membrane functionalization [19,20,23]. Through this approach, we sought to increase the probability of efficient NK–tumor engagement and thereby amplify downstream cytotoxic responses.

NK cells exert antitumor activities through coordinated recognition and cytotoxic execution mechanisms. Tumor cells are identified via missing-self or induced-self signals reflecting altered MHC class I expression or stress ligand upregulation. Upon this recognition, NK cells trigger apoptosis of cancer cells through FasL/TRAIL-mediated death receptor signaling and cytotoxic granule release. Meanwhile, IFN-γ secreted from NK cells primarily functions to modulate the tumor microenvironment and activate other immune cells [44]. These effector pathways are critically dependent on the stable formation of immunological synapses between NK cells and tumor targets [45]. Downregulation of activating ligands, alterations in tumor cell membrane composition, and spatial constraints within multicellular aggregates collectively reduce the probability of sustained synapse formation. Therefore, engineering strategies that enhance physical tumor recognition at the membrane level without compromising intrinsic cytotoxicity represent an attractive approach to improve NK cell efficacy [46]. To enhance the targeting ability of NK cells toward solid tumors, a series of cell surface engineering methods have been proposed to install tumor-targeting functional moieties onto cell membranes [47]. Among the available approaches, lipid-mediated hydrophobic insertion offers several distinct advantages compared to other cell surface modifications. Direct insertion of functional ligands to membrane proteins via covalent conjugation could provide permanent modification but risks perturbing the conformation of various membrane compartments and compromising cell viability [48]. Electrostatic adsorption or layer-by-layer coating are technically straightforward. However, the resulting multilayered coatings randomly cover cell surfaces and thereby hinder the accessibility and activity of signaling receptors and ligands [20]. In contrast, DSPE-PEG lipid anchors spontaneously intercalate into plasma membranes through hydrophobic insertion, enabling the rapid and efficient cell surface functionalization with tumor-targetable ligands while preserving membrane-presented receptor/ligand availability [38]. In the present study, we designed a PLE-Lipid conjugate to enhance the targeting capability of NK cells to ovarian cancer. The rational design of lipid-based functional biomaterials requires optimization of key structural parameters, particularly the lipid anchor and PEG spacer length. Previous studies have shown that two-tailed phospholipids, such as DSPE, exhibit superior membrane retention compared to cholesterol- or DMPE-based anchors [38]. In addition, our prior work identified PEG2k as the optimal spacer, providing balanced membrane retention, homogeneous surface distribution, and minimal internalization [37]. Based on these prior optimizations, we selected DSPE as the membrane anchoring lipid for its prolonged retention on NK cell membranes relative to other lipid candidates. PEG2k was incorporated as a spacer to reduce nonspecific internalization and maintain functional presentation of the targeting moiety. Importantly, this DSPE-PEG membrane engineering framework exhibits modular adaptability across different tumor types. For example, DSPE-PEG-hyaluronic acid constructs were successfully employed to target CD44-overexpressing pancreatic cancer cells, resulting in enhanced NK–tumor engagement and augmented cytotoxic efficacy [25]. In addition, a variety of ligand moieties, including peptide-based ligands (e.g., PD-L1-binding peptides and RGD peptides) [49,50], small molecules (e.g., folic acid and phenylboronic acid), and polysaccharides (e.g., hyaluronic acid), can be readily incorporated to target diverse tumor-specific markers [23]. Moreover, because membrane anchoring is driven by hydrophobic insertion of the lipid moiety, this strategy can be extended beyond immune cells to other membrane-based systems, such as exosomes, extracellular vesicles, and liposomes, further broadening its applicability [51,52]. Collectively, these features establish the DSPE-PEG platform as a versatile and broadly applicable system for enhancing immune cell targeting across diverse solid tumors. Collectively, these findings established the DSPE-PEG platform as a broadly applicable and modular system that is able to enhance immune cell targeting across diverse solid tumors [53].

Based on this strategy, we designed a PLE-Lipid conjugate composed of three functional modules: (1) DSPE as an anchor for stable membrane insertion, (2) PEG as a spacer, and (3) PLE as an ovarian tumor-interactive domain. This amphiphilic architecture was intended to achieve stable localization onto NK cell membranes while maintaining ligand accessibility and biological functionality (Scheme 1).

The PLE-Lipid conjugate was synthesized via EDC/NHS-mediated amide coupling between DSPE-PEG-COOH and PLE (Figure 1A). The successful formation of the conjugate was verified through ^1^H-NMR and FT-IR analyses (Figure 1B,C). In the ^1^H-NMR spectrum analysis [51], characteristic proton signals corresponding to PEG chains (δ ~3.5 ppm), DSPE lipid hydrocarbon chains (δ 1.05 to 1.18 ppm), and PLE segments (δ 2.05 to 2.18 ppm) were simultaneously observed, indicating the successful incorporation of DSPE-PEG and PLE within the final construct. FT-IR spectra further supported amide bond formation between DSPE-PEG and PLE, as evidenced by the appearance of characteristic amide peaks, including N-H stretching (~3282 cm^−1^) and amide I and II bands (~1647 and ~1546 cm^−1^), together with C-H stretching of lipid chains (~2917 cm^−1^). These spectral features collectively confirm the successful synthesis of the amphiphilic PLE-Lipid conjugate used for NK cell membrane engineering [24].

Figure 2 indicates the successful membrane insertion of PLE-Lipid. Fluorescence microscopic images of NK cells treated with PLE-Lipid-FL revealed membrane-localized fluorescence outlining the cellular perimeter, indicative of surface immobilization, rather than intracellular accumulation (Figure 2A,B). Flow cytometric analysis indicating a concentration-dependent increase in MFI supports controllable and tunable membrane functionalization [17]. Importantly, the gradual and proportional increase in signal across concentrations suggests the homogeneous insertion onto NK membranes rather than aggregation-mediated adsorption (Figure 2C). Such homogeneous insertion contrasts with direct covalent surface modification strategies. For example, maleimide–thiol-based conjugation approaches, such as those used to attach drug-loaded nanoparticles to CAR-engineered NK cells, relied on the distribution of surface-exposed thiols on membrane proteins, and, therefore, resulted in heterogeneous or patchy surface engineering [54]. In contrast, our DSPE-PEG lipid anchors intercalated directly into the phospholipid bilayer of cells and subsequently underwent lateral diffusion within the membrane plane, enabling more uniform and predictable surface functionalization while preserving membrane protein integrity. Temporal analysis further showed that fluorescence intensity remained near initial post-coating levels for the first 2 h and gradually declined thereafter, remaining detectable up to 17 h (Figure 2D). This dynamic detachment behavior reflects continuous membrane turnover and lipid exchange processes, leading to gradual loss of surface-bound conjugates. Importantly, despite partial coating detachment, sufficient surface anchoring of PLE-Lipid conjugates is maintained within a biologically relevant timeframe to support initial tumor targeting following systemic administration and subsequent NK cell–tumor interactions [55].

In addition to maintaining NK cell viability and proliferative capacity (Figure 3A), the preservation of intrinsic NK effector functions was also a critical parameter for the development of surface-engineered NK cells. One of the critical drawbacks in a material-mediated cellular membrane modification is the architectural hindrance to signal-binding membrane compartments. For example, in a single-cell surface encapsulation using LbL-based multilayer polymeric nano-shells, physical blocking of membrane receptor accessibility in HeLa cells and subsequently decreased antigen targeting were observed [56]. In contrast to this potential risk of receptor masking following surface engineering, our lipid-mediated surface engineering successfully preserved death receptor-mediated cytotoxic signaling in the NK–cancer interfaces. Flow cytometric analysis in Figure 3B,C revealed that the surface presentation levels of FasL and TRAIL remained unchanged following PLE-Lipid coating. Since both FasL and TRAIL engage FAS and DR4/DR5 on target cancer cells to induce apoptosis [57], these results demonstrate that the membrane insertion of PLE-Lipid biomaterials did not sterically hinder apoptotic ligand presentation. Consistent with this observation, functional killing assays of surface-engineered NK cells confirmed that baseline cytotoxic activity was preserved, as shown in Figure 4.

In NK cell killing mechanism, two primary functionalities of tumor recognition and cytotoxic efficacy are of importance. To enhance the target recognition ability of NK cells, we selected PLE as a targeting moiety that exploits characteristic features of tumor cell membranes, particularly those associated with ovarian cancer [29]. Most existing ligand-based targeting strategies rely on the overexpression of specific surface receptors [25,26,58], the levels of which can vary substantially across tumor types and between patients due to tumor heterogeneity, thereby limiting their targeting efficiency [59]. Conventional DSPE-PEG-based targeting strategies rely on specific ligand–receptor interactions, such as HA-CD44 or folic acid–folate receptor systems [25,26]. In contrast, PLE enables a receptor-independent tumor recognition strategy by interacting with cholesterol-rich lipid raft domains [30], which are frequently enriched in cancer cell membranes due to altered lipid metabolism [33]. Because this approach does not depend on specific receptor expression, it may provide a more broadly applicable targeting strategy for heterogeneous solid tumors (Figure 4A). This distinction highlights a conceptual shift from conventional receptor-targeting approaches toward membrane domain-targeting strategies in immune cell surface engineering. Ovarian cancer cells frequently overexpress mucins, such as MUC1 [60]. The repeating carboxylated units in PLE also confer mucoadhesive properties toward mucin-rich tumor membranes via transient multivalent interactions [35,61].

The effector–target conjugation assays in Figure 4B,C show that a significantly higher level of NK–tumor cluster formation was achieved in the PLE-NK cell group compared to the unmodified control NK cells (~1.6-fold increase after 30 min at a 1:1 E:T ratio). Importantly, when target OVCAR-3 cells were pre-treated with MβCD to deplete membrane cholesterol (Figure 4B), this enhancement was markedly attenuated, indicating that cholesterol-rich membrane domains contribute to PLE-mediated tumor recognition. In contrast, when fibroblast cells were used as target cells under the same experimental conditions, low levels of E:T cluster formation (~2%) were observed across all groups, which were markedly lower than those observed with ovarian cancer cells, indicating the minimal nonspecific interaction of PLE-NK cells with non-malignant cells. Although compared to the unmodified NK cells, the PLE-NK cells showed a slight increase in cluster formation (Figure 4C), the overall binding remained limited and did not translate into increased cytotoxic activity against fibroblast cells.

Following PLE-mediated ovarian cancer recognition, we sought to further evaluate the activation of NK cells by assessing both cytokine secretion and lytic granules associated with immune responses and cytotoxic activity [62]. Activated NK cells mediate antitumor activity through both immunomodulatory cytokine secretion and direct cytotoxic mechanisms involving cytotoxic granules [63]. In particular, TNF-α contributes to antitumor activity by inducing apoptotic signaling in tumor cells and enhancing local inflammatory responses [64], whereas IFN-γ acts as a central immunoregulatory cytokine that promotes immune cell recruitment and activation [65]. Granzyme B is a critical effector molecule secreted from activated NK cells that is responsible for inducing apoptosis in target cells by directly cleaving and activating effector caspases, such as caspase-3 [66,67].

Based on these mechanisms, we evaluated TNF-α, IFN-γ, and granzyme B secretion to assess the functional activation of PLE-NK cells (Figure 4D). As a result, increased engagement between NK cells and ovarian cancer cells (Figure 4B) was associated with significantly higher TNF-α secretion in PLE-NK cells compared to unmodified NK cells, along with elevated IFN-γ and granzyme B secretion (Figure 4D). Collectively, these findings resulted in approximately 2-fold higher specific lysis of OVCAR-3 cells in 4 h killing assays (Figure 4E). This 2-fold enhancement in cytotoxicity observed in our DSPE-PEG-based PLE system is meaningful when compared with existing NK cell engineering strategies. Antibody- or aptamer-mediated targeting approaches typically yield moderate improvements (~1.3–2-fold) by enhancing tumor recognition [51,68], while drug delivery-based surface engineering strategies achieve approximately ~1.5-fold enhancement through localized drug release [69]. Genetic engineering approaches, such as CAR-NK systems, can result in higher cytotoxicity (~2–3-fold), although some studies report comparable levels (~1.5–2-fold), depending on the model system [70]. Taken together, our PLE-based strategy demonstrates a level of cytotoxic enhancement that is comparable to existing approaches, despite its non-genetic and relatively simple design. Notably, as a receptor-independent, membrane-targeting strategy, it may offer broader applicability across heterogeneous tumor types, highlighting its potential as a complementary and scalable NK cell engineering platform. Furthermore, minimal lysis toward normal fibroblasts (Figure 4E) confirmed (1) tumor selectivity without non-specific hyperactivation of PLE-NK cells and (2) preferentially enhanced tumor cell engagement NK cell surface engineering.

Notably, enhanced cytotoxicity was also observed in 3D tumoroid models, where PLE-NK cells induced pronounced tumoroid disruption and tumor cell death (Figure 4F). As 3D architectures impose spatial constraints that limit immune cell infiltration and stable immunological synapse formation [71], improved killing efficacy in this structural setting further suggests functional relevance in ovarian tumors, which frequently disseminates as multicellular aggregates within the peritoneal cavity. Taken together, these results demonstrate the functional potential of the proposed surface engineering strategy under physiologically relevant conditions.

While NK-92MI cells were used in this study as a standardized model system, further validation in primary NK cells will be essential for clinical translation. Importantly, the proposed strategy is based on a non-genetic, lipid-mediated membrane insertion mechanism, suggesting its broad applicability to primary NK cells. However, differences in membrane dynamics and receptor expression profiles may require additional optimization of lipid composition and PEG design. In the present study, we demonstrated enhanced tumor recognition and anticancer efficacy of PLE-NK through NK cells mediated by interactions between membrane-decorated PLE and cholesterol-rich domains on tumor cells, in both 2D and 3D tumoroid models. While the enhanced tumor recognition and cytotoxicity observed in this study highlight the potential of PLE-NK cells, their in vivo therapeutic efficacy and safety remain to be fully validated. Future studies incorporating relevant in vivo models will be essential to assess tumor accumulation, therapeutic efficacy, and systemic toxicity.

In this study, we developed a lipid-mediated cellular membrane engineering strategy to enhance the recognition and cytotoxic efficacy of NK cells against ovarian cancer. By introducing PLE-Lipid conjugates onto the NK cell surface through lipid anchor-mediated hydrophobic and lipophilic insertion into the plasma membrane, we enabled rapid and stable membrane functionalization to target ovarian cancer. This approach allows efficient ex vivo cell surface engineering without genetic modification while preserving intrinsic NK cell viability and effector functions. Importantly, our PLE-Lipid-mediated surface engineering did not interfere with the primary effector functions of NK cells.

The engineered PLE-NK cells exhibited enhanced physical engagement with ovarian cancer cells, resulting in increased effector–target cluster formation and improved cytotoxic activity. We confirmed that this augmented recognition was associated with the dynamic interaction between NK cell membrane-presented PLE and cholesterol-rich domains on ovarian cancer cell membranes. Elimination of tumor membrane cholesterol significantly reduced NK–tumor cluster formation, confirming the role of cholesterol in mediating PLE-mediated tumor recognition. Following PLE-Lipid insertion, the membrane accessibility of FasL and TRAIL remained unchanged, indicating that extrinsic apoptotic signaling pathways and cytotoxic functions were preserved. Consistently, specifically facilitated tumor killing was observed in ovarian cancer cells without inducing nonspecific cytotoxicity toward normal fibroblasts.

In conclusion, our findings demonstrate that lipid biomaterial-mediated membrane engineering can effectively improve NK cell–tumor physical engagement and anticancer efficacy by modulating cell–cell interactions at the immunological synapse interfaces. The modular design of the PLE-Lipid conjugate provides a versatile strategy to functionalize immune cell surfaces with tumor-targeting ligands and may offer a broadly applicable framework to enhance immune cell-based therapies against solid tumors.

## Data Availability

The data that support the findings of this study are available from the corresponding author upon reasonable request.

## Abbreviations

The following abbreviations are used in this manuscript:

NK	Natural killer cells
PLE	Poly-L-glutamic acid
DSPE	1,2-distearoyl-sn-glycero-3-phosphoethanolamine
HGSOC	High-grade serous ovarian carcinoma
PEG	Polyethylene glycol
PLE-Lipid	Poly-L-glutamic acid–lipid conjugate
PLE-Lipid-FL	Fluorescently labeled poly-L-glutamic acid–lipid conjugate
PLE-NK	PLE-Lipid-coated NK cells
E:T	Effector-to-target
MFI	Mean fluorescence intensity
TME	Tumor microenvironment
OVCAR-3	Human ovarian carcinoma cell line
FITC	Fluorescein isothiocyanate
PBS	Phosphate-buffered saline
FBS	Fetal bovine serum
RIPA	Radioimmunoprecipitation assay buffer
WST-1	Water-soluble tetrazolium-1 assay
ANOVA	Analysis of variance
MβCD	Methyl-β-cyclodextrin
FasL	Fas ligand
TRAIL	Tumor necrosis factor-related apoptosis-inducing ligand
MUC1	Mucin 1
MUC16	Mucin 16
HA	Hyaluronic acid
FA	Folic acid
LBL	Layer-by-layer
CAR	Chimeric antigen receptor
NMR	Nuclear magnetic resonance
FT-IR	Fourier transform infrared spectroscopy
IFN-γ	Interferon-gamma
TNF-α	Tumor necrosis factor-alpha
